# XRCC1 Gene Polymorphisms and the Risk of Differentiated Thyroid Carcinoma (DTC): A Meta-Analysis of Case-Control Studies

**DOI:** 10.1371/journal.pone.0064851

**Published:** 2013-05-22

**Authors:** Yi Bao, Lei Jiang, Jue-Yu Zhou, Jun-Jie Zou, Jiao-Yang Zheng, Xiang-Fang Chen, Zhi-Min Liu, Yong-Quan Shi

**Affiliations:** 1 Department of Endocrinology, Changzheng Hospital, Second Military Medical University, Shanghai, China; 2 Department of Neurosurgery, Changzheng Hospital, Second Military Medical University, Shanghai, China; 3 Institute of Genetic Engineering, Southern Medical University, Guangzhou, China; The University of Texas M. D. Anderson Cancer Center, United States of America

## Abstract

**Background:**

Previous studies investigating the association between X-ray repair cross-complementing group 1 (XRCC1) polymorphisms and thyroid cancer risk have yielded inconsistent results. This meta-analysis was performed to derive a more precise estimation of the relationship between three XRCC1 polymorphisms and thyroid cancer risk.

**Methods/Principal Findings:**

PubMed and EMBASE database were systematically searched to identify relevant studies. 10 publications were selected for this meta-analysis, including 11 studies for Arg399Gln polymorphism (1726 cases and 3774 controls), 7 studies for Arg194Trp polymorphism (1037 cases and 2487 controls) and 8 studies for Arg280His polymorphism (1432 cases and 3356 controls). The results in total population did not show any significant association between these three polymorphisms and the risk of DTC for all genetic models. However, when stratified by ethnicity, the results showed that Arg280His polymorphism was associated with an increased risk of DTC among Caucasians (Arg/His vs. Arg/Arg: OR = 1.45, 95% CI = 1.09–1.93; dominant model: OR = 1.43, 95% CI = 1.08–1.89; additive model: OR = 1.38, 95% CI = 1.05–1.80), whereas individuals carrying Arg/His genotype have a significantly reduced risk of DTC among Asians (Arg/His vs. Arg/Arg: OR = 0.71, 95% CI = 0.51–0.98). We also detected that 399Gln variant allele carriers might presented an overall decreased risk of DTC in mixed population. Furthermore, subgroup analyses by histological subtype revealed that Arg194Trp polymorphism was significantly associated with reduced risk for papillary thyroid carcinoma (PTC) (dominant model: OR = 0.71, 95% CI = 0.50–0.99).

**Conclusions:**

This meta-analysis suggests that Arg280His polymorphism might contribute to the susceptibility of DTC among Caucasians, whereas it might provide protective effects in Asians against the risk of DTC. Additionally, our results support the protective role of Arg194Trp polymorphism in developing PTC, and show evidence of an association between Arg399Gln polymorphism and decreased risk of DTC in mixed population.

## Introduction

Thyroid cancer is the most prevalent endocrine malignancy with increasing incidence rates in recent years [1], [2]. It can be classified into four forms (papillary, follicular, medullary and anaplastic) based on different histopathological characteristics. Pathologically, papillary thyroid carcinoma (PTC) and follicular thyroid carcinoma (FTC) are termed differentiated thyroid carcinoma (DTC), accounting for approximately 90% of all thyroid malignancies. Although the exact etiology of thyroid cancer remains unknown, exposure to ionizing radiation is the only verified cause of thyroid carcinogenesis, especially radiation exposure during childhood or as a young adult [3], [4]. However, not all of those who have been exposed to ionizing radiation will develop thyroid cancer, and most patients do not have the history of radiation exposure, suggesting that host factors, including genetic polymorphisms, may have an impact on an individual's susceptibility to thyroid cancer.

DNA damage, caused by ionizing radiation, environmental toxins, and metabolic chemicals, can lead to gene mutations and genomic instability, which in turn may contribute to tumorigenesis. There are four major DNA repair pathways in mammals, including base excision repair (BER), nucleotide excision repair (NER), mismatch repair (MMR) and double strand break repair (DSBR) [5]. Among them, BER is the predominant DNA damage repair pathway for the processing of endogenous DNA lesions as well as damages produced during episodes of inflammation and exposures to ionizing radiation or a variety of chemical carcinogens [6]. At least 20 proteins are involved in BER pathway, including X-ray repair cross-complementing group 1 (XRCC1), apurinic/apyrimidinic endonuclease 1 (APE1), 8-oxoguanine DNA glycosylase 1 (OGG1), etc [7]. The mutations and single-nucleotide polymorphisms (SNPs) in corresponding genes may impair their repair or reversal capacity and increase the risk of cancer [6].

XRCC1 gene is an important component of the BER pathway encoding a scaffolding protein, which functions as a facilitator or coordinator in this pathway by directly interacting with poly (ADP-ribose) polymerase (PARP), DNA polymerase beta, and DNA ligase III [8], [9], [10]. Although numerous validated SNPs in XRCC1 gene have been identified in the dbSNP database (http://www.ncbi.nlm.nih.gov/SNP), only three of which are most widely investigated including Arg194Trp on exon 6 (rs1799782, C/T), Arg280His on exon 9 (rs25489, G/A), and Arg399Gln on exon 10 (rs25487, G/A) [11]. These XRCC1 polymorphisms may affect DNA repair capacity by changing interactions between XRCC1 protein and other proteins in BER pathway, and a large number of studies have focused on the relationship between XRCC1 polymorphisms and development of cancer in humans [12], [13], [14], [15].

Over the past decade, several epidemiological studies have reported the association regarding XRCC1 polymorphisms and thyroid cancer risk [16], [17], [18], [19], [20], [21], [22], [23], [24], [25], [26]. However, the results are to some extent divergent, but nevertheless intriguing. And majority of studies involved no more than a few hundred thyroid cancer cases, which may have been underpowered to detect a slight effect or may have generated a fluctuated risk estimate. So far, no quantitative summary of the evidence has ever been performed. To clarify the effect of XRCC1 polymorphisms (Arg399Gln, Arg280His, and Arg194Trp) on thyroid cancer risk, we carried out a meta-analysis of all eligible case-control studies.

## Materials and Methods

### Identification and selection of relevant studies

A comprehensive literature search was performed using the PubMed and EMBASE database to identify studies that evaluated the association between XRCC1 polymorphisms and the risk of thyroid cancer up to December 18, 2012 with the following terms and keywords: (XRCC1 or “X-ray repair cross-complementation group 1” or “DNA repair gene”), (“thyroid cancer” or “thyroid carcinoma”) and (polymorphism or variant or variation). The search was limited to human studies. In addition, references cited in the retrieved articles were reviewed to trace additional relevant studies missed by the searching.

### Inclusion criteria

The following inclusion criteria were used to select literatures for the meta-analysis: 1) a case–control study evaluating at least one polymorphism in the XRCC1 gene; 2) studies with full text articles; 3) sufficient data for estimating an odds ratio (OR) with 95% confidence interval (CI); 4) no overlapping data. If studies had the same or overlapping data, only the largest study should be included in the final analysis.

### Data extraction

Two investigators reviewed and extracted information from all eligible publications independently, according to the inclusion criteria listed above. For conflicting evaluation, a consensus was reached by discussion. The following data were collected from each study: first author, year of publication, country, ethnicity (categorized as Asian, Caucasian, or mixed descent), source of controls (population-based [PB] or hospital-based [HB] controls), genotyping method, numbers of cases and controls, genotype frequency of cases and controls, and the results of Hardy–Weinberg equilibrium (HWE) test.

### Statistical analysis

We first assessed HWE in the controls for each study using a web-based program (http://ihg2.helmholtz-muenchen.de/cgi-bin/hw/hwa1.pl) and a *P*<0.05 was considered as significant disequilibrium. The strength of association between these three XRCC1 polymorphisms (Arg399Gln, Arg194Trp and Arg280His) and thyroid cancer risk was measured by ORs with 95% CIs. The pooled ORs were performed for dominant model (aa+Aa vs. AA, a was for the minor allele and A was for the major allele), recessive model (aa vs. Aa+AA), codominant model (aa vs. AA, Aa vs. AA) and additive model (a vs. A), respectively. Heterogeneity assumption was checked by a chi-square-based *Q* test [27], and *I* ^2^ statistics was calculated to quantify the proportion of the total variation across studies due to heterogeneity [28]. The pooled ORs were calculated by a fixed-effects model (the Mantel-Haenszel method) when the *P* value>0.05 for the *Q* test which indicated a lack of heterogeneity among the studies [29]. Otherwise, a random-effects model (DerSimonian-Laird method) was used [30]. To explore the effect of heterogeneity among the studies on the conclusions of this meta-analysis, subgroup analyses were performed by ethnicity and histological subtype (papillary thyroid carcinoma and follicular thyroid carcinoma). Sensitivity analysis was performed by omitting each study in turn to assess the stability of results. The publication bias was diagnosed by the funnel plot, in which the standard error of log (OR) of each study was plotted against its log (OR). Funnel plot asymmetry was further assessed by the method of Egger's linear regression test (*P*<0.05 was considered a significant publication bias) [31]. All of the statistical analyses used in our meta-analysis were performed by STATA version 11.0 (Stata, College Station, TX, USA).

## Results

### Study characteristics

**Figure 1:**
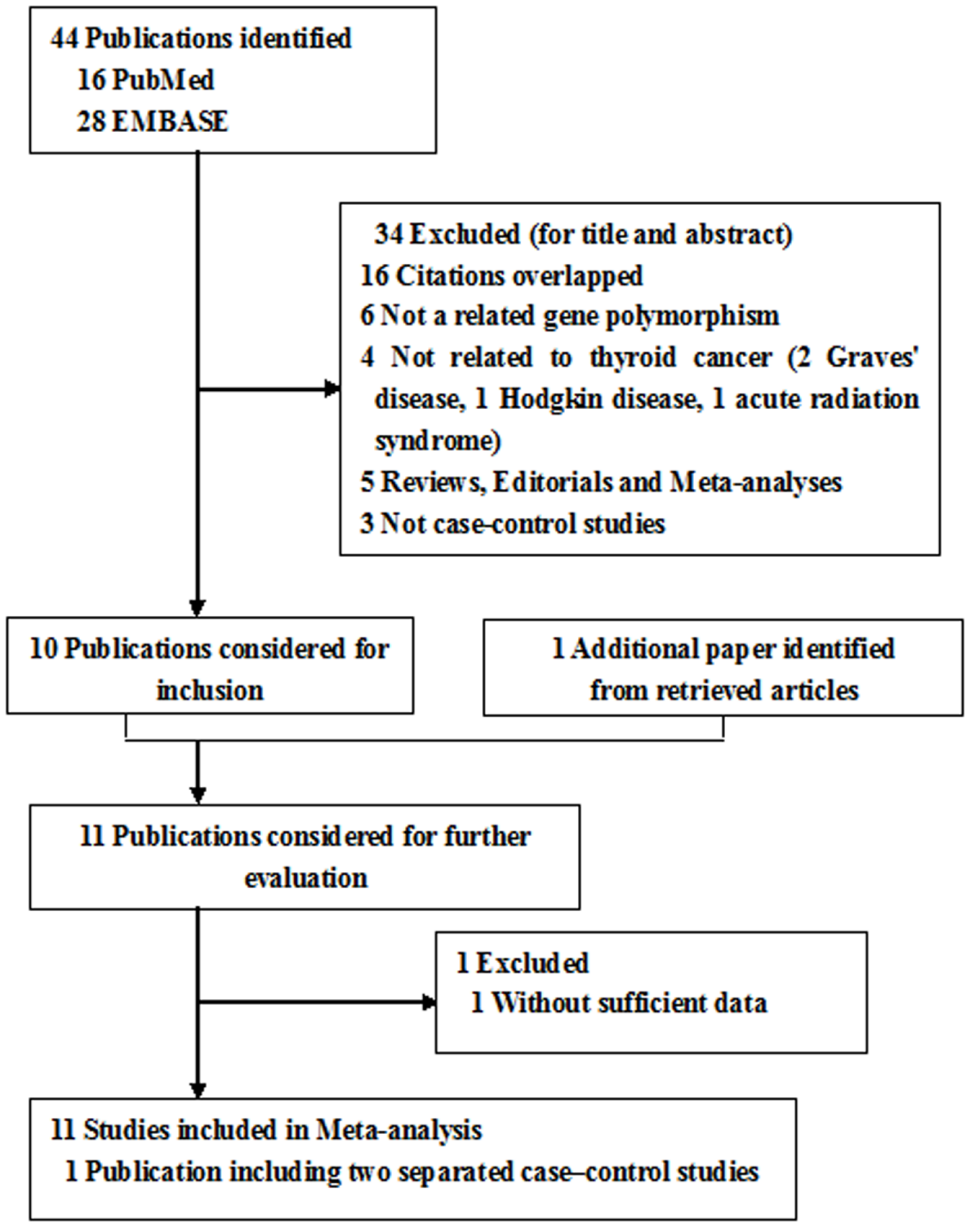
Flow diagram of included studies for this meta-analysis.

A total of eleven publications were preliminarily retrieved based on the inclusion criteria for risks of thyroid cancer related to the XRCC1 polymorphisms [16], [17], [18], [19], [20], [21], [22], [23], [24], [25], [26]. Among which, one was excluded because the data of genotyping distribution was missing [26], two separated case–control studies were included from the publication of Akulevich et al. [19] and were considered separately. And part of the data were analyzed only in dominant genetic model because the publication of Sigurdson et al. [21] only provide the limited genotyping information for two XRCC1 polymorphisms (Arg194Trp and Arg280His). Hence, there were 11 studies for Arg399Gln polymorphism (1726 cases and 3774 controls), 7 studies for Arg194Trp polymorphism (1037 cases and 2487 controls) and 8 studies for Arg280His polymorphism (1432 cases and 3356 controls). Our initial search and the process of study selection were summarized in Figure 1, while the main characteristics of included studies were listed in Table 1. And the study for Iran population was considered as Caucasian descent, but not Asian descent since the genotype distribution and allele frequency of controls of XRCC1 among Iran population seems more similar as Caucasians, as shown in the Table 2. Therefore, there were 5 studies of Caucasians [19], [22], [24], [25], 4 studies of Asians [16], [17], [18], [23] and 2 studies of mixed population [20], [21]. In addition, some of the studies provided genotype data for specific histological subtypes of thyroid cancer, such as PTC and FTC. Among them, six studies focused on PTC [16], [18], [19], [21], [23], and only the study by Santos et al. [25] on both PTC and FTC. The distribution of genotypes in the controls of each study was in agreement with Hardy–Weinberg equilibrium except for two studies (only for the Arg399Gln polymorphism) [21], [23]. Among these studies, 10 were hospital-based and only one was population-based. Most of the cases were confirmed histologically or pathologically.

**Table 1:** Characteristics of studies included in the meta-analysis.

Author	Year	Ethnicity	Region	SNPs studied	Genotyping	Control source	Cases/Controls
Santos	2012	Caucasian	Portugal	399, 194	PCR-RFLP	HB	109/217, 108/217
Fard-Esfahani	2011	Asian	Iran	399, 194, 280	PCR-RFLP	HB	155/190, 157/187, 170/193
Ryu	2011	Asian	Korea	399, 194	PCR-RFLP	HB	111/100, 111/100
García-Quispes	2011	Caucasian	Spain	399, 280	iPLEX Assay	HB	386/474,398/473
Akulevich-a	2009	Caucasian	Russia/Belarus	399, 280	PCR-RFLP	PB	123/197, 123/195
Akulevich-b	2009	Caucasian	Russia/Belarus	399, 280	PCR-RFLP	HB	132/398, 132/398
Ho	2009	Mixed	USA	399, 194, 280	PCR-RFLP	HB	251/503, 251/503, 251/503
Sigurdson	2009	Mixed	Kazakhstan	399, 194, 280	Taqman	HB	24/892, 25/906, 25/896
Chiang	2008	Asian	Taiwan	399, 194, 280	Taqman	HB	283/469, 283/469, 283/469
Siraj	2008	Asian	Saudi Arabia	399, 280	PCR-RFLP	HB	50/229, 50/229
Zhu	2004	Asian	China	399, 194	PCR-RFLP	HB	105/105, 105/105

Abbreviations: RFLP, restriction fragment length polymorphism; TaqMan, real-time TaqMan analysis; iPLEX Assay: Increased Plexing Efficiency.

and Flexibility for MassARRAY platform; **PB, population-based; HB, hospital-based**.

**Table 2:** Genotype distribution of XRCC1 polymorphisms used in the meta-analysis.

Polymorphism	First author	Year	Ethnicity	Control source	Sample size (case/control)		Case			Control				HWE	MAF
							AA	Aa	aa	AA	Aa		aa		
**Arg399Gln**	Santos	2012	Caucasian	HB	106	217	44	50	12	87	105		25	0.43	0.36
	Fard-Esfahani	2011	Caucasian	HB	155	190	78	60	17	83	87		20	0.69	0.33
	Ryu	2011	Asian	HB	111	100	87	17	7	72	19		9	<0.01	0.19
	García-Quispes	2011	Caucasian	HB	386	474	153	186	47	196	212		66	0.48	0.36
	Akulevich-a	2009	Caucasian	PB	123	197	55	50	18	75	100		22	0.18	0.37
	Akulevich-b	2009	Caucasian	HB	132	398	65	53	14	158	193		47	0.30	0.36
	Ho	2009	Mixed	HB	251	503	133	99	19	220	216		67	0.23	0.35
	Sigurdson	2009	Mixed	HB	24	892	12	10	2	460	343		89	0.036	0.29
	Chiang	2008	Asian	HB	283	469	150	110	23	277	165		27	0.71	0.23
	Zhu	2004	Asian	HB	105	105	49	44	12	57	45		3	0.09	0.24
	Siraj	2008	Asian	HB	50	229	35	13	2	142	72		15	0.16	0.22
**Arg194Trp**	Santos	2012	Caucasian	HB	105	217	95	8	2	196	21		0	0.45	0.05
	Fard-Esfahani	2011	Caucasian	HB	157	187	136	18	3	166	20		1	0.64	0.06
	Ryu	2011	Asian	HB	111	100	59	43	9	37	49		14	0.73	0.39
	Ho	2009	Mixed	HB	251	503	203	45	3	433	69		1	0.31	0.07
	Sigurdson	2009	Mixed	HB	25	906	20	5		665	241			–	–
	Chiang	2008	Asian	HB	283	469	127	119	37	234	199	36		0.48	0.29
	Zhu	2004	Asian	HB	105	105	50	52	3	48	51	6		0.11	0.30
**Arg280His**	Fard-Esfahani	2011	Caucasian	HB	170	193	146	23	1	173	18	2		0.07	0.06
	García-Quispes	2011	Caucasian	HB	398	473	337	58	3	426	44	3		0.12	0.05
	Akulevich-a	2009	Caucasian	PB	123	195	113	10	0	176	19	0		0.47	0.05
	Akulevich-b	2009	Caucasian	HB	132	398	117	15	0	366	32	0		0.40	0.04
	Ho	2009	Mixed	HB	251	503	229	22	0	453	50	0		0.24	0.05
	Sigurdson	2009	Mixed	HB	25	896	24	1		800	96			–	–
	Chiang	2008	Asian	HB	283	469	224	54	5	349	113	7		0.53	0.14
	Siraj	2008	Asian	HB	50	229	33	12	5	129	79	21		0.09	0.26

Abbreviations: HWE, Hardy-Weinberg equilibrium; MAF, minor allele frequency; A, the major allele; a, the minor allele.

### Quantitative synthesis

**Figure 2:**
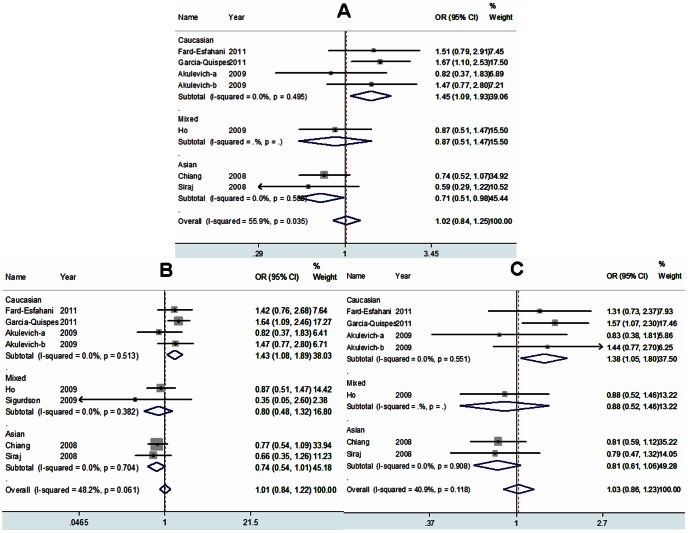
Forest plots of ORs with 95% CI for XRCC1 Arg280His polymorphism and the risk of DTC observed in subgroup analyses by ethnicity (fixed effects). The center of each square represents the OR, the area of the square is the number of sample and thus the weight used in the meta-analysis, and the horizontal line indicates the 95%CI. (A) Arg/His vs. Arg/Arg. (B) Dominant model. (C) Additive model.

**Figure 3:**
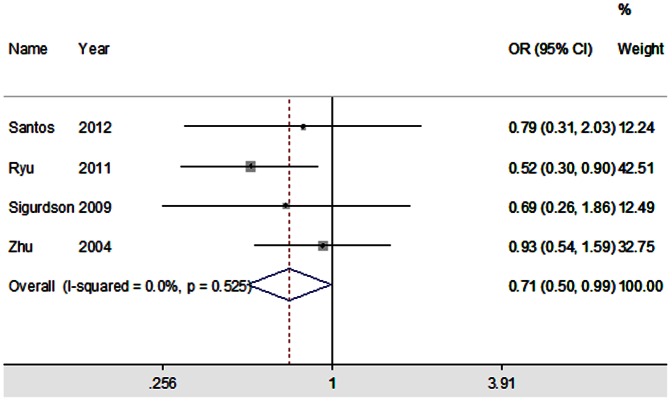
Forest plot of ORs with 95% CI for XRCC1 Arg194Trp polymorphism and the risk of PTC under dominant model. The center of each square represents the OR, the area of the square is the number of sample and thus the weight used in the meta-analysis, and the horizontal line indicates the 95%CI.

Table 3 summarized the main results of the meta-analysis for XRCC1 polymorphisms. For Arg399Gln polymorphism, there was no statistically significant difference between this polymorphism and the risk of DTC in all genetic models when all eligible studies were pooled together. Similarly, the combined results did not showed any association between Arg194Trp/Arg280His polymorphisms and the risk of DTC for all genetic models. However, when stratified by ethnicity, the results showed that Arg/His genotype was associated with an increased risk of DTC among Caucasians (Arg/His vs. Arg/Arg: OR = 1.45, 95% CI = 1.09–1.93; dominant model: OR = 1.43, 95% CI = 1.08–1.89; additive model: OR = 1.38, 95% CI = 1.05–1.80), whereas individuals carrying Arg/His genotype have a significantly reduced risk of DTC among Asians (Arg/His vs. Arg/Arg: OR = 0.71, 95% CI = 0.51–0.98). (Figure 2) And carriers of the 399Gln variant allele might have a decreased risk of DTC in mixed population (dominant model: OR = 0.73, 95% CI = 0.55–0.97; recessive model: OR = 0.56, 95% CI = 0.34–0.93; Gln/Gln vs. Arg/Arg: OR = 0.50, 95% CI = 0.30–0.85; additive model: OR = 0.73, 95% CI = 0.59–0.92). We also detected that the Trp allele of Arg194Trp polymorphism was significantly associated with increased risk of DTC in mixed population (additive model: OR = 1.49, 95% CI = 1.02–2.17). Furthermore, stratified analyses by histological subtype showed that Arg194Trp polymorphism was significantly associated with reduced risk for PTC in dominant model (OR = 0.71, 95% CI = 0.50–0.99). (Figure 3) However, no evidence of significant association between Arg399Gln/Arg280His polymorphisms and the risk of PTC was found. In addition, subgroup analysis by the source of controls was not performed due to the limited data for population-based studies.

**Table 3:** Results of meta-analysis for Arg399Gln, Arg194Trp and Arg280His polymorphisms and the risk of DTC and PTC.

Genetic model		Recessive model			Dominant model			Homozygote			Heterozygote			Additive model		
Arg399Gln	n	Gln/Gln vs. Arg/Gln + Arg/Arg			Gln/Gln + Arg/Gln vs. Arg/Arg			Gln/Gln vs. Arg/Arg			Arg/Gln vs. Arg/Arg			Gln vs. Arg		
		OR(95%CI)	*P*_h_	*I* ^2^(%)	OR(95%CI)	*P*_h_	*I* ^2^(%)	OR(95%CI)	*P*_h_	*I* ^2^(%)	OR(95%CI)	*P*_h_	*I* ^2^(%)	OR(95%CI)	*P*_h_	*I* ^2^(%)
Total	11(1726/3774)	0.94(0.77–1.15)	0.163	29.7	0.91(0.80–1.03)	0.088	39.1	0.90(0.73–1.10)	0.091	38.7	0.91(0.80–1.04)	0.239	21.5	0.93(0.81–1.07)	0.031	49.5
Ethnicity																
Caucasian	5(902/1476)	0.97(0.75–1.25)	0.828	0.0	0.88(0.74–1.04)	0.335	12.4	0.90(0.69–1.19)	0.941	0.0	0.87(0.73–1.04)	0.187	35.1	0.92(0.82–1.05)	0.743	0.0
Asian	4(549/903)	1.35(0.88–2.08)	0.108	50.7	1.11(0.89–1.39)	0.156	42.6	1.38(0.89–2.14)	0.073	57.0	1.08(0.85–1.37)	0.401	0.0	1.13(0.94–1.36)	0.051	61.5
Mixed	2(275/1395)	**0.56(0.34–0.93)**	**0.588**	**0.0**	**0.73(0.55–0.97)**	**0.326**	**0.0**	**0.50(0.30–0.85)**	**0.460**	**0.0**	0.80(0.59–1.07)	0.403	0.0	**0.73(0.59–0.92)**	**0.308**	**3.6**
Histological subtype																
PTC	7(623/2138)	1.13(0.82–1.57)	0.323	14.1	0.85(0.70–1.04)	0.382	5.9	1.02(0.73–1.43)	0.238	25.1	0.82(0.66–1.01)	0.620	0.0	0.94(0.80–1.09)	0.188	31.4

Arg194Trp	n	Trp/Trp vs. Arg/Trp + Arg/Arg			Trp/Trp + Arg/Trp vs. Arg/Arg			Trp/Trp vs. Arg/Arg			Arg/Trp vs. Arg/Arg			Trp vs. Arg		
		OR(95%CI)	*P*_h_	*I* ^2^(%)	OR(95%CI)	*P*_h_	*I* ^2^(%)	OR(95%CI)	*P*_h_	*I* ^2^(%)	OR(95%CI)	*P*_h_	*I* ^2^(%)	OR(95%CI)	*P*_h_	*I* ^2^(%)
Total	7(1037/2487)	1.42(0.62–3.24)	0.041	56.9	1.07(0.89–1.29)	0.089	45.4	1.43(0.55–3.69)	0.013	65.2	1.03(0.85–1.26)	0.209	30.2	1.08(0.82–1.43)	0.018	63.2
Ethnicity																
Caucasian	2(262/404)	5.43(0.90–32.79)	0.583	0.0	1.12(0.68–1.84)	0.677	0.0	5.43(0.90–32.82)	0.594	0.0	0.96(0.57–1.63)	0.546	0.0	1.27(0.80–2.02)	0.827	0.0
Asian	3(499/674)	0.88(0.33–2.32)	0.025	72.9	0.87(0.52–1.44)	0.026	72.7	0.78(0.24–2.52)	0.007	79.8	0.95(0.74–1.21)	0.118	53.2	0.90(0.57–1.43)	0.006	80.7
Mixed	2(276/1409)	6.07(0.63–58.68)	–	–	1.30(0.90–1.87)	0.168	47.4	6.40(0.66–61.90)	–	–	1.39(0.92–2.10)	–	–	**1.49(1.02–2.17)**	–	–
Histological subtype																
PTC	4(318/1328)	0.71(0.35–1.40)	0.112	54.3	**0.71(0.50–0.99)**	**0.525**	**0.0**	0.60(0.30–1.23)	0.088	58.8	0.71(0.49–1.03)	0.314	13.8	0.76(0.58–1.01)	0.302	16.5

Arg280His	n	His/His vs. Arg/His + Arg/Arg			His/His + Arg/His vs. Arg/Arg			His/His vs. Arg/Arg			Arg/His vs. Arg/Arg			His vs. Arg		
		OR(95%CI)	*P*_h_	*I* ^2^(%)	OR(95%CI)	*P*_h_	*I* ^2^(%)	OR(95%CI)	*P*_h_	*I* ^2^(%)	OR(95%CI)	*P*_h_	*I* ^2^(%)	OR(95%CI)	*P*_h_	*I* ^2^(%)
Total	8(1432/3356)	1.08(0.56–2.10)	0.957	0.0	1.01(0.84–1.22)	0.061	48.2	1.00(0.51–1.96)	0.958	0.0	1.04(0.75–1.42)	0.035	55.9	1.03(0.86–1.23)	0.118	40.9
Ethnicity																
Caucasian	4(823/1259)	0.94(0.25–3.50)	0.614	0.0	**1.43(1.08–1.89)**	**0.513**	**0.0**	0.99(0.26–3.70)	0.608	0.0	**1.45(1.09–1.93)**	**0.495**	**0.0**	**1.38(1.05–1.80)**	0.551	0.0
Asian	2(333/698)	1.14(0.53–2.45)	0.924	0.0	0.74(0.54–1.01)	0.704	0.0	1.01(0.46–2.19)	0.823	0.0	**0.71(0.51–0.98)**	**0.582**	**0.0**	0.81(0.61–1.06)	0.908	0.0
Mixed	2(276/1399)	–	–	–	0.80(0.48–1.32)	0.382	0.0	–	–	–	0.87(0.51–1.47)	–	–	0.88(0.52–1.46)	–	–
Histological subtype																
PTC	4(330/1718)	1.10(0.39–3.07)	–	–	0.87(0.59–1.29)	0.263	24.7	0.93(0.33–2.65)	–	–	0.91(0.61–1.38)	0.173	43.0	0.95(0.67–1.36)	0.316	13.2

*P*_h_ *P* values for heterogeneity from *Q* test. Random-effects model was used when *P* value for heterogeneity test<0.05; otherwise, fixed-model was used.

### Test of heterogeneity and sensitivity analyses

Significant heterogeneity was detected for Arg194Trp polymorphism (recessive model comparison, additive model comparison and homozygote comparison), Arg399Gln polymorphism (additive model comparison) and Arg280His polymorphism (heterozygote comparison). To explore the potential sources of heterogeneity, we assessed the pooled ORs under all comparisons via subgroup and sensitivity analyses. We found that ethnicity (χ^2^ = 10.90, df = 2, *P* = 0.004 for Arg280His polymorphism; χ^2^ = 9.01, df = 2, *P* = 0.011 for Arg399Gln polymorphism) but not the source of controls (χ^2^ = 0.34, df = 1, *P* = 0.560 for Arg280His polymorphism; χ^2^ = 0.01, df = 1, *P* = 0.920 for Arg399Gln polymorphism) contributed to substantial heterogeneity for Arg280His/Arg399Gln polymorphisms. Although there were two studies deviated from HWE for Arg399Gln polymorphism, the corresponding pooled ORs were not materially altered by including or not including these studies (data not shown). In addition, we evaluated the influence of each individual study on the overall ORs for Arg194Trp/Arg280His polymorphisms. And the results showed the pooled ORs of these two polymorphisms were not materially altered by the results of any individual study, suggesting that the results of this meta-analysis are credible (data also not shown).

### Publication bias

**Figure 4:**
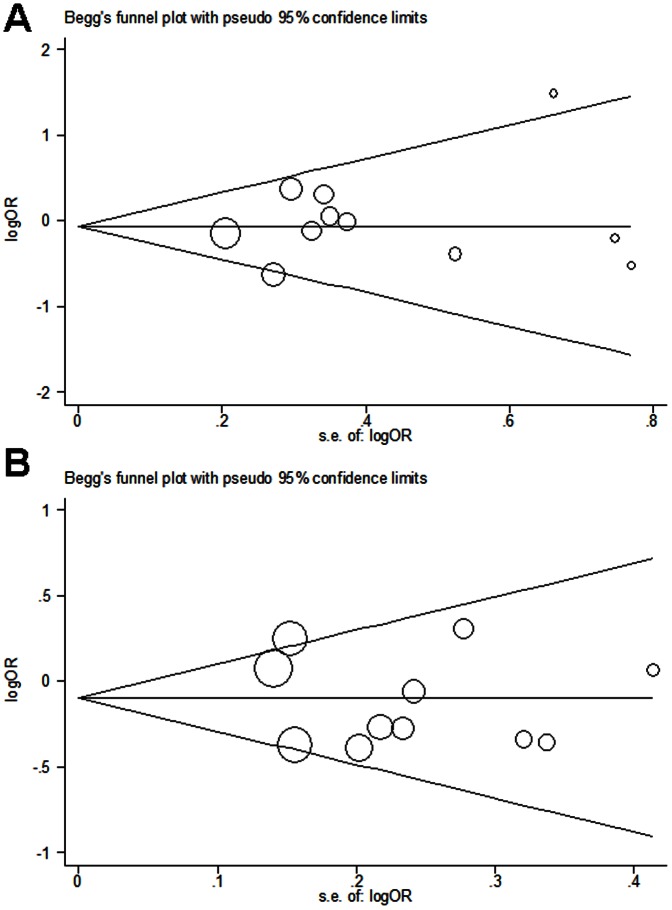
Begg's funnel plots of Arg399Gln polymorphism and the risk of DTC for publication bias test. Each point represents a separate study for the indicated association. Log (OR), natural logarithm of OR. Horizontal line, mean effect size. (A) Recessive model. (B) Dominant model.

We performed Begg's funnel plot and Egger's test to assess the publication bias in this meta-analysis. The shapes of the funnel plots did not reveal any evidence of obvious asymmetry (Figure 4). The results of Egger's test did not suggest any evidence of publication bias for Arg399Gln polymorphism (*P* = 0.602 for Gln/Gln vs. Arg/Arg, *P* = 0.342 for Arg/Gln vs. Arg/Arg, *P* = 0.534 for dominant model, *P* = 0.473 for recessive model and *P* = 0.798 for additive model), Arg194Trp polymorphism (*P* = 0.818 for Trp/Trp vs. Arg/Arg, *P* = 0.306 for Arg/Trp vs. Arg/Arg, *P* = 0.234 for dominant model, *P* = 0.754 for recessive model and *P* = 0.636 for additive model) and Arg280His polymorphism (*P* = 0.588 for His/His vs. Arg/Arg, *P* = 0.992 for Arg/His vs. Arg/Arg, *P* = 0.656 for dominant model, *P* = 0.236 for recessive model and *P* = 0.821 for additive model), respectively.

## Discussion

The DNA repair system plays a pivotal role in maintaining the genome integrity and stability through the reversal of DNA damage. Genetic variations in DNA repair genes are thought to modify DNA repair capacity and suggested to be related to cancer risk [32], [33], [34]. The XRCC1, encoding an important scaffolding protein that participate in the BER pathway, has multiple roles in repairing DNA base damage and single-strand DNA breaks. More than 300 validated SNPs have been identified in this gene, of which, Arg399Gln, Arg194Trp and Arg280His polymorphisms were most extensively studied [11]. These non-conservative amino acid alterations may alter XRCC1 function and contribute to the risk of cancers [35]. To date, several studies have been conducted to evaluate the association between XRCC1 polymorphisms and thyroid cancer risk in different ethnic populations, but the results remain conflicting rather than conclusive [16], [17], [18], [19], [20], [21], [22], [23], [24], [25], [26].

Previously, two studies by Akulevich et al. [19] and Ho et al. [20], respectively, reported that Arg399Gln polymorphism was associated with decreased risk of DTC and PTC, whereas Arg399Gln variant genotype carriers presented an increased risk of PTC in a Chinese study [16]. However, more studies did not support an association between Arg399Gln polymorphism and thyroid cancer risk [17], [21], [22], [23], [24], [25]. Furthermore, Ho et al. [20] reported that Arg194Trp variant homozygote genotype was associated with increased risk of DTC, in agreement with the conclusion by Chiang et al. [17]. In contrast, it was reported that the heterozygous genotype was significantly associated with a decreased risk of PTC in a Korean population [23]. And other studies did not reveal statistically significant association regarding this polymorphism and thyroid cancer [16], [21], [24], [25]. As for Arg280His polymorphism, Garcia-Quispes et al. [22] addressed the variant genotype showed a highly increased risk for DTC among Caucasian, while two studies by Akulevich et al. [19] and Fard-Esfahani et al. [24] found similar trends toward having DTC, but statistical significance was not attained. Moreover, another two studies suggested that Arg280His heterozygous genotype might provide protective effects against the risk of thyroid cancer among Asians, but this also did not reach statistical significance [17], [18]. The discrepancies across these studies motivated the present meta-analysis.

More importantly, many systematic reviews and meta-analyses have addressed the association of XRCC1 polymorphisms with various cancers [36], [37], [38], [39], [40], [41], [42], but have not evaluated the association between these polymorphisms and thyroid cancer. In other words, this is the first meta-analysis undertaken so far of the largest and most comprehensive assessment for the relationship between XRCC1 polymorphisms and the susceptibility to thyroid cancer. Our meta-analysis did not show any significant association between these three polymorphisms (Arg399Gln, Arg194Trp, and Arg280His) and the risk of DTC in the total population for all genetic models. Interestingly, in the subgroup analysis by ethnicity, the results indicated that Arg280His polymorphism was associated with a significantly increased risk of DTC among Caucasians under dominant genetic model, additive genetic model and heterozygote comparison, whereas heterozygote Arg/His genotype might provide protective effects in Asians against the risk of DTC. We also detected that individuals harboring variant allele of Arg399Gln polymorphism might have a decreased risk of DTC in mixed population, but not in Caucasians or Asians. In contrast, the Arg194Trp variant allele carriers might have an increased risk of DTC in mixed population, but not in Caucasians or Asians. Several factors may contribute to different roles of the same polymorphism in cancer susceptibility among different ethnic populations. Above all, cancer is a complicated multi–genetic disease, and different genetic backgrounds may contribute to the discrepancy. Another explanation may be that the low penetrance genetic effects of single polymorphism usually depends on interaction with other polymorphisms and/or a particular environmental exposure including tobacco smoke, ionizing radiation, dietary and other lifestyles. Besides, other factors such as selection bias and different matching criteria may also result in the difference. However, our results for Arg194Trp polymorphism should be interpreted with caution because only one study of mixed population was included for the analysis of the additive model. Considering the limited numbers of studies, in the future, larger studies are warranted to validate possible ethnic differences in the effect of these polymorphisms on the risk of thyroid cancer.

Strikingly, stratified analysis by histological classification of thyroid cancer revealed that Arg194Trp polymorphism was significantly associated with reduced risk for PTC in dominant model. On the contrary, Arg399Gln and Arg280His polymorphisms did not appear to have an overall influence on the susceptibility to PTC. Actually, only four studies explored the possible association between Arg194Trp polymorphism and the risk of PTC. In line with our findings, Ryu et al. [23] reported that Arg/Trp genotype carriers presented an overall decreased risk of PTC, in Asians (OR = 0.55, 95% CI = 0.31–0.98). Similarly, other three studies suggested that the Trp allele might provide protective effects against developing PTC in different populations, but statistical significance was not attained [16], [21], [25]. Although our results strongly suggested its role in the development of PTC, it should not be excluded that this effect could be related to sample size. Thus, given the existence of etiologic heterogeneity within different histological types of thyroid cancer, subtype-specific studies including adequate numbers of cases are needed to verify its potential usefulness as a predictive biomarker of genetic susceptibility to different subtypes of thyroid cancer.

Although we have put considerable effort and resources into testing possible association between XRCC1 polymorphisms and thyroid cancer risk, there are still some limitations in this meta-analysis. First, the number of published studies was not sufficiently large for a comprehensive analysis, especially for subgroup analyses by ethnicity and histological subtype. Stratified analyses by histological subtype were only performed for PTC, but not for FTC, because only one study [25] reported separate genotype frequency for FTC. Second, gene–gene, gene–environment, or even different polymorphism loci of the XRCC1 gene interactions were not examined in this meta-analysis because of the insufficient data. Actually, several studies demonstrated the effect of gene-gene interactions between XRCC1 polymorphisms and other genes in DNA repair pathway on cancer risk [17], [43], [44], [45]. Also, the studies by Chiang et al. [17] and Ho et al. [20] found that multiple haplotypes of XRCC1 polymorphisms were associated with a significantly increased risk of DTC. Therefore, it is of particular interest to elucidate the utility of XRCC1 haplotypes in predicting the risk of thyroid cancer. Third, our results were based on single-factor estimates without adjustment for other risk factors such as age, gender, radiation exposure dose, smoking status, drinking consumption, obesity, or other variables, which might have caused confounding bias. For instance, the genetic risk in the study by Chiang et al. [17] was more predominant in DTC cases that showed neck lymph node (LN) metastasis. Thus, a more precise analysis should be conducted if detailed individual data are available. Last but not the least, some inevitable publication bias might exist in the results because only published studies were retrieved although the funnel plot and Egger's test indicated no remarkable publication bias.

In conclusion, this meta-analysis suggests that Arg280His polymorphism might contribute to the susceptibility of DTC among Caucasians, whereas it might provide protective effects in Asians against the risk of DTC. Additionally, our results support the protective role of Arg194Trp polymorphism in developing PTC, and show evidence of an association between Arg399Gln polymorphism and decreased risk of DTC in mixed population. Nevertheless, large-scale, well-designed and population-based studies are needed to investigate haplotypes, gene–gene, and gene–environment interactions on these polymorphisms and the risk of thyroid cancer and its histological subtypes in an ethnicity specific population, which may eventually lead to better comprehensive understanding of the possible roles in thyroid tumorigenesis.

## Supporting Information

Table S1 **PRISMA 2009 Checklist for this Meta-analysis.**
(DOC)

## References

[1] Chen AY, Jemal A, Ward EM (2009) Increasing incidence of differentiated thyroid cancer in the United States, 1988-2005. Cancer 115: 3801–3807. 19598221 10.1002/cncr.24416

[2] Jemal A, Siegel R, Ward E, Hao Y, Xu J, et al (2008) Cancer statistics, 2008. CA Cancer J Clin 58: 71–96. 18287387 10.3322/CA.2007.0010

[3] Tronko MD, Howe GR, Bogdanova TI, Bouville AC, Epstein OV, et al (2006) A cohort study of thyroid cancer and other thyroid diseases after the chornobyl accident: thyroid cancer in Ukraine detected during first screening. J Natl Cancer Inst 98: 897–903. 16818853 10.1093/jnci/djj244

[4] Xiong P, Zheng R, Wang LE, Bondy ML, Shen H, et al (2005) A pilot case-control study of gamma-radiation sensitivity and risk of papillary thyroid cancer. Thyroid 15: 94–99. 15753665 10.1089/thy.2005.15.94

[5] Wood RD, Mitchell M, Sgouros J, Lindahl T (2001) Human DNA repair genes. Science 291: 1284–1289. 11181991 10.1126/science.1056154

[6] Wallace SS, Murphy DL, Sweasy JB (2012) Base excision repair and cancer. Cancer Lett 327: 73–89. 22252118 10.1016/j.canlet.2011.12.038 PMC3361536

[7] Hu Z, Ma H, Chen F, Wei Q, Shen H (2005) XRCC1 polymorphisms and cancer risk: a meta-analysis of 38 case-control studies. Cancer Epidemiol Biomarkers Prev 14: 1810–1818. 16030121 10.1158/1055-9965.EPI-04-0793

[8] Caldecott KW, Tucker JD, Stanker LH, Thompson LH (1995) Characterization of the XRCC1-DNA ligase III complex in vitro and its absence from mutant hamster cells. Nucleic Acids Res 23: 4836–4843. 8532526 10.1093/nar/23.23.4836 PMC307472

[9] Kubota Y, Nash RA, Klungland A, Schar P, Barnes DE, et al (1996) Reconstitution of DNA base excision-repair with purified human proteins: interaction between DNA polymerase beta and the XRCC1 protein. EMBO J 15: 6662–6670. 8978692 PMC452490

[10] Caldecott KW, McKeown CK, Tucker JD, Ljungquist S, Thompson LH (1994) An interaction between the mammalian DNA repair protein XRCC1 and DNA ligase III. Mol Cell Biol 14: 68–76. 8264637 10.1128/mcb.14.1.68-76.1994 PMC358357

[11] Shen MR, Jones IM, Mohrenweiser H (1998) Nonconservative amino acid substitution variants exist at polymorphic frequency in DNA repair genes in healthy humans. Cancer Res 58: 604–608. 9485007

[12] Tae K, Lee HS, Park BJ, Park CW, Kim KR, et al (2004) Association of DNA repair gene XRCC1 polymorphisms with head and neck cancer in Korean population. Int J Cancer 111: 805–808. 15252855 10.1002/ijc.20338

[13] Wang Y, Yang H, Li H, Li L, Wang H, et al (2009) Association between X-ray repair cross complementing group 1 codon 399 and 194 polymorphisms and lung cancer risk: a meta-analysis. Cancer Lett 285: 134–140. 19481337 10.1016/j.canlet.2009.05.005

[14] Shen H, Xu Y, Qian Y, Yu R, Qin Y, et al (2000) Polymorphisms of the DNA repair gene XRCC1 and risk of gastric cancer in a Chinese population. Int J Cancer 88: 601–606. 11058877 10.1002/1097-0215(20001115)88:4<601::aid-ijc13>3.0.co;2-c

[15] Xie T, Wang ZG, Zhang JL, Liu H (2012) X-ray repair cross-complementing group 1 polymorphisms and hepatocellular carcinoma: A meta-analysis. World J Gastroenterol 18: 4207–4214. 22919255 10.3748/wjg.v18.i31.4207 PMC3422803

[16] Zhu QX, Bian JC, Shen Q, Jiang F, Tang HW, et al (2004) [Genetic polymorphisms in X-ray repair cross-complementing gene 1 and susceptibility to papillary thyroid carcinoma]. Zhonghua Liu Xing Bing Xue Za Zhi 25: 702–705. 15555397

[17] Chiang FY, Wu CW, Hsiao PJ, Kuo WR, Lee KW, et al (2008) Association between polymorphisms in DNA base excision repair genes XRCC1, APE1, and ADPRT and differentiated thyroid carcinoma. Clin Cancer Res 14: 5919–5924. 18779313 10.1158/1078-0432.CCR-08-0906

[18] Siraj AK, Al-Rasheed M, Ibrahim M, Siddiqui K, Al-Dayel F, et al (2008) RAD52 polymorphisms contribute to the development of papillary thyroid cancer susceptibility in Middle Eastern population. J Endocrinol Invest 31: 893–899. 19092295 10.1007/BF03346438

[19] Akulevich NM, Saenko VA, Rogounovitch TI, Drozd VM, Lushnikov EF, et al (2009) Polymorphisms of DNA damage response genes in radiation-related and sporadic papillary thyroid carcinoma. Endocr Relat Cancer 16: 491–503. 19286843 10.1677/ERC-08-0336

[20] Ho T, Li G, Lu J, Zhao C, Wei Q, et al (2009) Association of XRCC1 polymorphisms and risk of differentiated thyroid carcinoma: a case-control analysis. Thyroid 19: 129–135. 19191745 10.1089/thy.2008.0153

[21] Sigurdson AJ, Land CE, Bhatti P, Pineda M, Brenner A, et al (2009) Thyroid nodules, polymorphic variants in DNA repair and RET-related genes, and interaction with ionizing radiation exposure from nuclear tests in Kazakhstan. Radiat Res 171: 77–88. 19138047 10.1667/RR1327.1 PMC2875679

[22] Garcia-Quispes WA, Perez-Machado G, Akdi A, Pastor S, Galofre P, et al (2011) Association studies of OGG1, XRCC1, XRCC2 and XRCC3 polymorphisms with differentiated thyroid cancer. Mutat Res 709–710: 67–72. 10.1016/j.mrfmmm.2011.03.003 21414327

[23] Ryu RA, Tae K, Min HJ, Jeong JH, Cho SH, et al (2011) XRCC1 polymorphisms and risk of papillary thyroid carcinoma in a Korean sample. J Korean Med Sci 26: 991–995. 21860547 10.3346/jkms.2011.26.8.991 PMC3154355

[24] Fard-Esfahani P, Fard-Esfahani A, Fayaz S, Ghanbarzadeh B, Saidi P, et al (2011) Association of Arg194Trp, Arg280His and Arg399Gln polymorphisms in X-ray repair cross-complementing group 1 gene and risk of differentiated thyroid carcinoma in Iran. Iran Biomed J 15: 73–78. 21987112 PMC3639746

[25] Santos LS, Branco SC, Silva SN, Azevedo AP, Gil OM, et al (2012) Polymorphisms in base excision repair genes and thyroid cancer risk. Oncol Rep 28: 1859–1868. 22922830 10.3892/or.2012.1975

[26] Neta G, Brenner AV, Sturgis EM, Pfeiffer RM, Hutchinson AA, et al (2011) Common genetic variants related to genomic integrity and risk of papillary thyroid cancer. Carcinogenesis 32: 1231–1237. 21642358 10.1093/carcin/bgr100 PMC3166197

[27] Cochran W (1954) The combination of estimates from different experiments. Biometrics 10: 101–129.

[28] Higgins JP, Thompson SG, Deeks JJ, Altman DG (2003) Measuring inconsistency in meta-analyses. BMJ 327: 557–560. 12958120 10.1136/bmj.327.7414.557 PMC192859

[29] Mantel N, Haenszel W (1959) Statistical aspects of the analysis of data from retrospective studies of disease. J Natl Cancer Inst 22: 719–748. 13655060

[30] DerSimonian R, Laird N (1986) Meta-analysis in clinical trials. Control Clin Trials 7: 177–188. 3802833 10.1016/0197-2456(86)90046-2

[31] Egger M, Davey Smith G, Schneider M, Minder C (1997) Bias in meta-analysis detected by a simple, graphical test. BMJ 315: 629–634. 9310563 10.1136/bmj.315.7109.629 PMC2127453

[32] Alberg AJ, Jorgensen TJ, Ruczinski I, Wheless L, Shugart YY, et al.. (2012) DNA repair gene variants in relation to overall cancer risk: a population-based study. Carcinogenesis. 10.1093/carcin/bgs304 PMC3534189 23027618

[33] Hung RJ, Hall J, Brennan P, Boffetta P (2005) Genetic polymorphisms in the base excision repair pathway and cancer risk: a HuGE review. Am J Epidemiol 162: 925–942. 16221808 10.1093/aje/kwi318

[34] Goode EL, Ulrich CM, Potter JD (2002) Polymorphisms in DNA repair genes and associations with cancer risk. Cancer Epidemiol Biomarkers Prev 11: 1513–1530. 12496039

[35] Monaco R, Rosal R, Dolan MA, Pincus MR, Brandt-Rauf PW (2007) Conformational effects of a common codon 399 polymorphism on the BRCT1 domain of the XRCC1 protein. Protein J 26: 541–546. 17899335 10.1007/s10930-007-9095-y

[36] Dai L, Wang K, Zhang J, Lv Q, Wu X, et al (2009) XRCC1 gene polymorphisms and esophageal squamous cell carcinoma risk in Chinese population: A meta-analysis of case-control studies. Int J Cancer 125: 1102–1109. 19444915 10.1002/ijc.24446

[37] Wang R, Hu X, Zhou Y, Feng Q, Su L, et al.. (2012) XRCC1 Arg399Gln and Arg194Trp polymorphisms in childhood acute lymphoblastic leukemia risk: a meta-analysis. Leuk Lymphoma. 10.3109/10428194.2012.704031 22712837

[38] Chen B, Zhou Y, Yang P, Wu XT (2012) Polymorphisms of XRCC1 and gastric cancer susceptibility: a meta-analysis. Mol Biol Rep 39: 1305–1313. 21604176 10.1007/s11033-011-0863-6

[39] Dai L, Duan F, Wang P, Song C, Wang K, et al (2012) XRCC1 gene polymorphisms and lung cancer susceptibility: a meta-analysis of 44 case-control studies. Mol Biol Rep 39: 9535–9547. 22729882 10.1007/s11033-012-1818-2

[40] Huang J, Zhang J, Zhao Y, Liao B, Liu J, et al (2011) The Arg194Trp polymorphism in the XRCC1 gene and cancer risk in Chinese Mainland population: a meta-analysis. Mol Biol Rep 38: 4565–4573. 21499756 10.1007/s11033-010-0588-y

[41] Li Y, Liu F, Tan SQ, Wang Y, Li SW (2012) X-Ray Repair Cross-Complementing Group 1 (XRCC1) Genetic Polymorphisms and Cervical Cancer Risk: A HuGE Systematic Review and Meta-Analysis. PLoS One 7: e44441. 22984511 10.1371/journal.pone.0044441 PMC3440401

[42] Wang L, Yin F, Xu X, Hu X, Zhao D (2012) X-ray repair cross-complementing group 1 (XRCC1) genetic polymorphisms and risk of childhood acute lymphoblastic leukemia: a meta-analysis. PLoS One 7: e34897. 22529951 10.1371/journal.pone.0034897 PMC3329555

[43] Kiuru A, Lindholm C, Heinavaara S, Ilus T, Jokinen P, et al (2008) XRCC1 and XRCC3 variants and risk of glioma and meningioma. J Neurooncol 88: 135–142. 18330515 10.1007/s11060-008-9556-y

[44] Zhang X, Miao X, Liang G, Hao B, Wang Y, et al (2005) Polymorphisms in DNA base excision repair genes ADPRT and XRCC1 and risk of lung cancer. Cancer Res 65: 722–726. 15705867

[45] Chacko P, Rajan B, Joseph T, Mathew BS, Pillai MR (2005) Polymorphisms in DNA repair gene XRCC1 and increased genetic susceptibility to breast cancer. Breast Cancer Res Treat 89: 15–21. 15666192 10.1007/s10549-004-1004-x

